# Opinion leaders and changes over time: a survey

**DOI:** 10.1186/1748-5908-6-117

**Published:** 2011-10-11

**Authors:** Gaby Doumit, Frances C Wright, Ian D Graham, Andrew Smith, Jeremy Grimshaw

**Affiliations:** 1 Institute of Dermatology and Plastic Surgery, Cleveland Clinic, Cleveland, OH, USA; 2 Department of Surgery-General Surgery, Sunnybrook Health Sciences Centre, Toronto, Canada; 3 Knowledge Translation and Public Outreach Portfolio, Canadian Institutes of Health Research, Ottawa, Canada; 4 Clinical Epidemiology Program, Ottawa Health Research Institute, Ottawa, Canada

## Abstract

**Background:**

Opinion leaders represent one way to disseminate new knowledge and influence the practice behaviors of physicians. This study explored the stability of opinion leaders over time, whether opinion leaders were polymorphic (*i.e.*, influencing multiple practice areas) or monomorphic (*i.e.*, influencing one practice area), and reach of opinion leaders in their local network.

**Methods:**

We surveyed surgeons and pathologists in Ontario to identify opinion leaders for colorectal cancer in 2003 and 2005 and to identify opinion leaders for breast cancer in 2005. We explored whether opinion leaders for colorectal cancer identified in 2003 were re-identified in 2005. We examined whether opinion leaders were considered polymorphic (nominated in 2005 as opinion leaders for both colorectal and breast cancer) or monomorphic (nominated in 2005 for only one condition). Social-network mapping was used to identify the number of local colleagues identifying opinion leaders.

**Results:**

Response rates for surgeons were 41% (2003) and 40% (2005); response rates for pathologists were 42% (2003) and 37% (2005). Four (25%) of the surgical opinion leaders identified in 2003 for colorectal cancer were re-identified in 2005. No pathology opinion leaders for colorectal cancer were identified in both 2003 and 2005. Only 29% of surgical opinion leaders and 17% of pathology opinion leaders identified in the 2005 survey were considered influential for both colorectal cancer and breast cancer. Social-network mapping revealed that only a limited number of general surgeons (12%) or pathologists (7%) were connected to the social networks of identified opinion leaders.

**Conclusions:**

Opinion leaders identified in this study were not stable over a two-year time period and generally appear to be monomorphic, with clearly demarcated areas of expertise and limited spheres of influence. These findings may limit the practicability of routinely using opinion leaders to influence practice.

## Background

Faced with consistent evidence about evidence-practice gaps, there is continued policy and research interest in strategies to promote evidence uptake to improve quality of care and patient safety. Early diffusion of innovation research highlighted the importance of influential individuals ("opinion leaders") within communities in promoting the rapid uptake and spread of innovations [1,2]. Rogers acknowledged that opinion leaders play a critical role in shepherding an innovation to that point at which a critical number of members of a social network have adopted the innovation such that further increases in the adoption become self-sustained [3]. These findings led to attempts to identify and recruit opinion leaders to promote evidence uptake.

There are four approaches to identifying opinion leaders: sociometric methods, key-informant methods, self-designating methods, and observation [3]. Sociometric methods involve extensive analyses of leadership nominations within members of a peer group and were employed in the present study. The majority of studies of opinion leaders in healthcare have used the Hiss instrument, a sociometric survey[4,5]. The Hiss instrument was developed based upon interviews with family doctors in Michigan that identified three characteristics of opinion leaders, namely that they encourage learning and enjoy sharing their knowledge, are clinical experts considered up-to-date, and treat others as equals[5-8]. It asks respondents to identify individuals who have each of these characteristics separately; individuals are considered opinion leaders if they are identified as having all three characteristics. The Hiss instrument has been used widely in dissemination and implementation research. Grimshaw and colleagues tested the convergent validity of the Hiss instrument and observed that identified opinion leaders were more likely than other respondents to possess hypothesized characteristics of opinion leaders (identified from diffusion of innovations and social influence theories)[9]. In a recent systematic review, 9 out of 12 randomized trials of opinion leadership used the Hiss instrument to identify opinion leaders [4]; all demonstrated short-term improvements in quality of care. While the use of opinion leaders appears to be a potentially useful dissemination and implementation strategy, there remain many important unanswered questions. First, the stability over time of perceptions about who is considered an opinion leader within a community has never been assessed. Second, it is important to know whether those considered opinion leaders are perceived to exert influence regarding multiple practice areas (polymorphic) or a single practice area pertaining to a particular disease (monomorphic). Third, the extent of influence of opinion leaders has rarely been described or mapped using social-network analysis.

In this study, we have built upon the work of Wright *et al.*, who surveyed general surgeons and pathologists in Ontario to determine who were the opinion leaders for colorectal cancer in 2003[5]. They surveyed all eligible general surgeons (n = 594) and pathologists (n = 358) in Ontario. Return rates were 41% for surgeons and 42% for pathologists [5]. No opinion leaders were identified in 39 of 81 (48%) hospitals. In the remaining hospitals, 16 surgeon and 6 pathologist local opinion leaders were identified. We resurveyed the same Ontario surgeons and pathologists in 2005 to determine if opinion leaders remain stable over time, whether opinion leaders are polymorphic or monomorphic, and to determine the reach of opinion leaders' influence using social-network mapping.

## Methods

This study is embedded in a randomized trial [10] comparing the influence of formal continuing medical education given by a provincial expert with or without reinforcement by local opinion leaders on the optimization of lymph node assessment in colorectal cancer (ISRCTN56824239). For the trial, surgeon and pathologist opinion leaders for colorectal cancer were identified using the Hiss survey instrument in 2003.

In 2005 we resurveyed the same pathologists and surgeons using the Hiss instrument to identify opinion leaders for the management of colorectal and breast cancer. The survey also asked about demographic information, including gender, age, numbers of years in practice, practice location, nature of practice, and estimated percentage of their clinical volume that related to colorectal cancer and breast cancer (Additional file 1, Additional file 2).

### Survey samples

To generate the mailing list for the 2005 survey, the 2003 physician survey list was reviewed. Physicians who declined to participate in the 2003 survey were excluded (n = 118), as were physicians whose clinical practice did not include any oncology (*e.g.*, vascular, pediatric, and cardio-thoracic surgeons), were retired, were not presently practicing in Ontario, had died, or if their practice no longer included colorectal cancer management (n = 43). In total, the 2005 survey was mailed to 480 general surgeons and 311 pathologists. Physicians were invited to return the survey using an enclosed stamped addressed envelope or a return fax number. An additional three mailings were completed.

If surveys were returned with an incorrect address and if we were unable to identify the correct address by searching the Royal College of Physicians and Surgeons of Canada online directory, Royal College of Physicians and Surgeons of Canada website, 411 Canada website, and the worldwide web, then these physicians were excluded.

Physicians were identified as opinion leaders if they were nominated as knowledgeable, as an educator, and as humanistic by at least one respondent.

### Opinion leader stability over time

We determined the proportion of colorectal cancer opinion leaders who were identified in the 2003 survey [5] that were re-identified as colorectal cancer opinion leaders in the 2005 survey.

### Polymorphism or monomorphism of opinion leaders

Monomorphism describes the situation where an opinion leader is considered influential in only one clinical area. Polymorphism represents a condition where an opinion leader is considered influential in multiple clinical areas. In this survey, if a physician was nominated as an opinion leader for either colorectal cancer or breast cancer, they were considered to be monomorphic but if they were nominated as an opinion leader for both colorectal cancer and breast cancer, then they were considered to be polymorphic.

### Assessment of opinion leaders' influence using social-network analysis

Social-networks plots were used to descriptively map the findings. Social-networks plots map the relationships (ties) between individuals (nodes) in a particular social network [11]. The nodes in the present network represent the survey respondents and opinion leaders, while the ties between the nodes illustrate the relationships among them.

The social-networks plots for the 2003 and 2005 surveys are made up of the general surgeon and pathologist respondents to the 2003 and 2005 surveys, respectively, and their nominated opinion leaders for colorectal cancer. They are tied together by the surgeons' and pathologists' relationships to their nominated colorectal cancer opinion leader.

Plotting social networks provides a visual representation of the relationships between respondents and identified opinion leaders. Analysis of the surgeons' and pathologists' social networks will help expose the pattern of links and help draw inferences about the social structure within which opinion leaders are embedded. The number of links between respondents and opinion leaders reflect the number of respondents who identified opinion leaders in their network. The strength of impact of an opinion leader within his or her medical community will come from the degree to which the opinion leader is at the center of many relationships. The more connected the individuals in the network, the more likely the information will readily diffuse [3].

### Statistical analysis

Analysis was performed using the SAS 9.1 statistical software package (SAS Institute, Inc., Cary, NC, USA). A chi-square or Fisher exact test was used to evaluate differences between categorical variables, and a student *t*-test was used to evaluate differences between means for continuous data. Medians were compared with the Wilcoxon two-sample test. Statistical significance was defined as *p *< .05, and all tests of significance were two tailed. In addition, PowerPoint^® ^(Microsoft Corporation, Redmond, WA, USA) was used to plot the social networks of general surgeons and pathologists.

Ethical approval for the project was obtained from the Ottawa Hospital Research Ethics Board.

## Results

### Survey returns

After four mailings, 37.2% (111/298) of eligible pathologists and 39.8% (177/445) of eligible general surgeons returned completed 2005 surveys.

Of the 152 pathologist respondents in 2003, 86 responded to the 2005 survey, for a recurrent response rate of 57%. For general surgeons, the recurrent response rate was 56%. There are no statistical differences between both groups of physicians regarding survey response (chi-square degrees of freedom = 1, 0.53; *p *= .47) and recurrent response ratio (chi-square degrees of freedom = 1, 0.01; *p *= .91). Thirty-one physicians returned a blank survey, which were considered to be refusals. When addresses of physicians in 2005 were compared to their addresses in 2003, there was a 3.3% and 5.5% change in pathologists' and general surgeons' addresses, respectively.

### Demographic characteristics of 2005 respondents

Fifty-nine percent of pathologists and 87% of general surgeons were male. There are significantly more males among general surgeons (*p *< .0001). The mean age in years for pathologists and general surgeons was 51.8 and 50.9, respectively. Colorectal cancer made up 10% and 15% of the pathologist and general surgeon respondents' clinical volume, respectively (*p *< .001). Breast cancer consisted of 10% of the pathologist and general surgeon respondents' estimated median clinical volume. Approximately 80% of pathologist and general surgeon respondents worked in urban centers.

### Demographic characteristics of 2005 nonrespondents

Four hundred and fifty-five physicians did not respond to our survey despite four mail outs. When sex, age, and mean years of clinical practices of nonrespondents were compared to respondents, there were no statistically significant differences.

### Opinion leaders' identification

#### Colorectal cancer

Physicians nominated by their community colleagues as knowledgeable, educators, and humanistic and whose advice they valued on colorectal cancer were considered opinion leaders for colorectal cancer. In the 2005 survey, 6 pathologists and 17 general surgeons were nominated as opinion leaders for colorectal cancer. Pathologist opinion leaders for colorectal cancer had a mean age of 50.7 years and were 67% male. They had on average 17.5 years of clinical experience, and colorectal cancer made up 17.5% of their clinical practice. Most of them (83%) worked in urban centers. Of the surgical opinion leaders for colorectal cancer, 76% were male and the mean age was 45 years. They had a mean of 12.5 years of clinical practice, and colorectal cancer made up 30% of their practice. Fifty-nine percent of the surgical opinion leaders worked in urban centers. No statistical difference in all characteristics for colorectal cancer-specific opinion leaders was reached between pathologists and general surgeons (.12 ≤ *p *≤ .63). In addition, there was no statistical difference between opinion leaders for colorectal cancer and survey participants on all characteristics examined (.08 ≤ *p *≤ .94).

#### Breast cancer

In the 2005 survey, 13 surgical and 1 pathologist opinion leaders for breast cancer were identified.

#### Stability over time of opinion leaders for colorectal cancer

No opinion leaders identified by pathologists in 2003 were re-identified in 2005. Five pathologists not identified as opinion leaders in the 2003 survey were identified as such in the 2005 survey. Four (25%) of the 16 opinion leaders identified in 2003 by general surgeons were re-identified in 2005 by the same respondents. Eight surgeons were newly identified in the 2005 survey.

#### Monomorphism or polymorphism of opinion leaders

In the 2005 survey, 13 surgical opinion leaders for breast cancer were identified. Of the 13 surgeons nominated as opinion leaders for breast cancer, five were also nominated as opinion leaders for colorectal cancer (29%). Pathologists identified only one opinion leader for breast cancer who was also an opinion leader for colorectal cancer (17%).

#### Mapping social networks for opinion leaders in colorectal cancer

**Figure 1:**
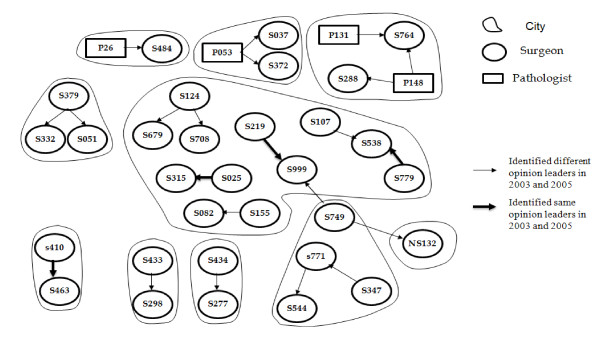
**Colorectal cancer surgeons' and pathologists' social network in 2003**. Only 13% (31/236) of surgeon and 3% (4/152) of pathologists' respondents to the 2003 survey are linked to the colorectal cancer opinion-leader social network in Ontario

**Figure 2:**
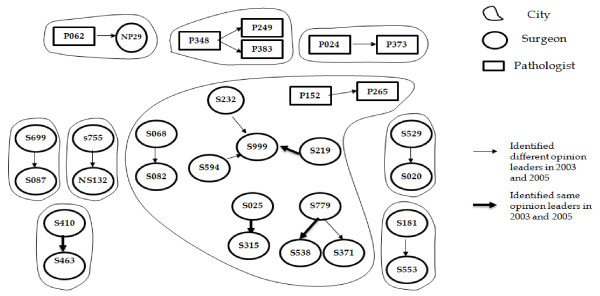
**Colorectal cancer surgeons' and pathologists' social network in 2005**. Only 12% (21/177) of surgeon and 7% (8/111) of pathologists respondents to the 2005 survey are linked to the colorectal cancer opinion-leader social network in Ontario

There was also a scarcity of links among the opinion leaders for colorectal cancer and the survey respondents when the social-network analysis was completed for both 2003 and 2005. Furthermore, a limited number of general surgeons (2003 survey: 31/236 = 13%; 2005 survey: 21/177 = 12%) and pathologists (2003 survey: 4/152 = 3%; 2005 survey: 8/111 = 7%) were connected to the networks (Figures 1 and 2).

## Discussion

### Summary of key findings

This study in two professional groups has demonstrated that opinion leaders did not remain the same over the two-year study period, that opinion leaders were perceived by the community to have very clearly demarcated areas of expertise and not be polymorphic, and that the sphere of influence attributed to opinion leaders in the fields of surgery and pathology is not extensive.

We found that none of the pathology opinion leaders and only 25% of the surgical opinion leaders for colorectal cancer who were identified in 2003 were re-identified by their peers in 2005. Further, eight surgeons and five pathologists were newly identified as opinion leaders in 2005. Together, these findings suggest the fluidity of opinion leaders and social networks.

These results imply that opinion leaders identified by the Hiss criteria need to be re-identified at least every two years if an opinion leader's influence is to be used in a knowledge-translation intervention. We are not entirely clear why perceptions of who are opinion leaders are so transient. It may be that an opinion leader's influence simply does not last very long. Alternatively, perhaps the Hiss opinion-leader identification instrument is not reliable. Interestingly, the test-retest reliability for the Hiss instrument has never been assessed in previous research. Hence, it is possible that this Hiss instrument will yield different opinion leaders on successive identification surveys. It is also possible that the formal identification of an opinion leader interferes with the opinion leader's future influence. Previous research has reported concerns about potential changes in the relationship between opinion leaders and other physicians in their networks once such individuals have been formally identified and subsequently trained to disseminate evidence-based research[12,13]. Locock (2001) has commented that there is a difficult balance to be struck for opinion leaders--their enthusiasm for a project may win others over and result in beneficial practice changes in some, but equally, this enthusiasm may single out such individuals as too different or radical and thereby undermine local influence. So by trying to formalize the role of opinion leaders as an educational channel, there is a danger that their previous informal influence may be lost [12].

The monomorphic nature of opinion leaders has been previously described in qualitative studies but not, as far as we are aware, in quantitative studies for this group of specialists [10,14]. In this study, only 29% of surgical opinion leaders and 17% of pathology opinion leaders were considered influential for both colorectal cancer and breast cancer. This has important practical implications for knowledge-translation activities involving opinion leaders in that interventions targeting different clinical conditions will require repeated identification surveys to identify relevant local opinion leaders.

Networks within which physicians are embedded have been shown to have an important influence on their attitudes and healthcare behaviors [15]. Marsden and Friedkin have stated that "the proximity of two actors in social networks is associated with the occurrence of interpersonal influence between the actors," where influence refers not just to conscious attempts to change behavior by using power or persuasion but also involves unconscious processes such as imitation, contagion, or comparison [16]. Analysis of the surgeons' and pathologists' social networks in the present study involved mapping relationships between survey responders and opinion leaders in order to expose the pattern of links and to help draw inferences about the social structure within which opinion leaders are embedded. General surgeons in Ontario appear to be more connected to their provincial colleagues than their counterparts in pathology. This observation suggests that general surgeons might be more primed for an opinion leader intervention than would pathologists. However, in Wright *et al.*'s (2004) intervention, it appeared that the influence of the local opinion leader had little impact on the number of lymph nodes assessed, which could be explained by their limited social network [10].

For the present work, there are a number of limitations. The first limitation was the low response rates, which lead to concerns about the generalizability of conclusions. However, our response rates were similar to response rates reported for other surveys using the Hiss instrument within both randomized trials and observational studies. While it is always preferable to see higher response rates, rates of this magnitude are acceptable for mail-out surveys, even with multiple reminders, so our response rates were expected and were consistent with those achieved in the 2003 survey. Concerns about nonresponse bias were attenuated by the observed lack of difference between participants and nonparticipants (in the second survey) with respect to sex, age, and clinical experience. The second limitation was that 5% of pathologists and 3% of surgeons changed hospitals between 2003 and 2005 and may have disrupted local social networks. However, as the number of address changes encountered appears to be low, we believe that the impact of such misclassification bias is likely to be minimal. Furthermore, by limiting the population used in the 2005 survey to the 2003 cohort of physicians, we think that contamination of our study population by incoming new specialists who were not exposed to the trial intervention was reduced. Finally, turnover of surgeons and pathologists is inevitable in the real world. A third limitation of the study is the reliability of the survey results, which is difficult to assess as no test-retest for the Hiss instrument has previously been performed.

We suggest that further research on the opinion-leader concept should focus on determining the reliability of the Hiss instrument as an opinion-leader identification tool. At the present, there is no way of determining whether the inability to replicate the results of the 2003 Hiss instrument survey by Wright *et al. *over two years' time is due to the short half-life of the opinion leaders or whether it is due to low reliability of the Hiss instrument. Furthermore, most research that has been concerned with opinion-leader selection and effectiveness in improving health professionals' behavior has not explored the extent of the reach of social networks within which the opinion leaders are embedded, which could be an important factor in the effectiveness of opinion-leader interventions.

## Conclusion

In conclusion, we have found that, for two professional groups in Ontario, opinion leaders did not remain the same over a two-year time period. Opinion leaders were perceived to have very clearly demarcated areas of expertise (*i.e.*, not polymorphic) and/or have limited spheres of influence by the respondents of this survey.

## Competing interests

The authors declare that they have no competing interests.

## Authors' contributions

GD participated in the design of the study, carried out the 2005 survey, performed the statistical analysis, and drafted the manuscript. FCW carried out the 2003 survey, participated in the design of the study, and helped to draft the manuscript. IDG participated in the study's design and coordination and helped to draft the manuscript. AS participated in the 2003 survey, participated in the design of the study, and helped to draft the manuscript. JG participated in the study's design and coordination and helped to draft the manuscript. JG was the senior research supervisor.

All authors read and approved the final manuscript.

## Supplementary Material

Additional file 1 **Hiss survey for pathologists**. The Hiss survey instrument used to identify opinion leaders for the management of colorectal and breast cancer among pathologists. Click here for file

Additional file 2 **Hiss survey for general surgeons**. The Hiss survey instrument used to identify opinion leaders for the management of colorectal and breast cancer among general surgeons. Click here for file
