# Supplementary material for: Opinion leaders and changes over time: a survey
Source: Implement Sci. 2011 Oct 11;6:117. doi: 10.1186/1748-5908-6-117 (PMC3205036; doi:10.1186/1748-5908-6-117)
Supplement: Additional file 2 — Hiss survey for general surgeons. The Hiss survey instrument used to identify opinion leaders for the management of colorectal and breast cancer among general surgeons. [file 1748-5908-6-117-S2.DOC]

***(Please Print)***

Please read the following items and think about the physicians in your community. If possible, please list up to two physicians in your community who best fit each description.

Your collaboration is **vital**. I sincerely thank you for your help and time. The entire survey should take about three minutes to complete.

***NOTE: You may include the same physician in more than one section and you may include your own name in any section that you feel is appropriate.***

### I. PHYSICIANS AS EDUCATORS

These physicians convey information in ways that lead to learning. They express themselves clearly and to the point. They provide practical information first and then an explanation or rationale if time allows. They take the time to answer you completely and do not leave you feeling that they were too busy to answer your inquiry. They enjoy and are willing to share any knowledge that they have.

Name: ____________________________ Name:____________________________

**II. PHYSICIANS AS KNOWLEDGEABLE PRACTITIONERS.**

These are physicians who like to teach. They seem always up-to-date, demonstrate a command of recent medical knowledge and a high level of clinical expertise.

Name: ____________________________ Name:____________________________

**III. PHYSICIANS AS CARING PROFESSIONALS**

These are physicians whom we might call real humanists. They treat people as equals; they listen, communicate well and never talk down to others even when helping them.

Name: ____________________________ Name:____________________________

**IV. GENERAL SURGEONS WHOSE ADVICE YOU VALUE ON COLORECTAL CANCER**

Name: ________________________Name:________________________

**V. GENERAL SURGEONS WHOSE ADVICE YOU VALUE ON BREAST CANCER**

Name: __________________________ Name:_________________________

1. **DESIGNATED LEADERS IN YOUR COMMUNITY**

E.g. - General Surgery Division Head.

Name: _________________________________________________________________

**Would you mind telling us a little about yourself?**

Gender: Female  Male  Age: ______ Number of Years in Practice: _____

Practice Location: Rural  Urban  Remote

Nature of your Practice (e.g. General Surgery, subspecialty surgery): ______________________________________________________________________

Estimated percentage of your clinical volume that relates to breast cancer: _____

Estimated percentage of your clinical volume that relates to colorectal cancer: _____
